# Resting Energy Expenditure Prediction in Recreational Athletes of 18–35 Years: Confirmation of Cunningham Equation and an Improved Weight-Based Alternative

**DOI:** 10.1371/journal.pone.0108460

**Published:** 2014-10-02

**Authors:** Twan ten Haaf, Peter J. M. Weijs

**Affiliations:** 1 Department of Nutrition & Dietetics, School of Sports and Nutrition, Amsterdam University of Applied Sciences, Amsterdam, The Netherlands; 2 Department of Nutrition & Dietetics, Internal Medicine, VU University Medical Center, Amsterdam, The Netherlands; 3 EMGO+ Institute for Health and Care Research, VU University Medical Center, Amsterdam, The Netherlands; Faculty of Biology, Spain

## Abstract

**Introduction:**

Resting energy expenditure (REE) is expected to be higher in athletes because of their relatively high fat free mass (FFM). Therefore, REE predictive equation for recreational athletes may be required. The aim of this study was to validate existing REE predictive equations and to develop a new recreational athlete specific equation.

**Methods:**

90 (53M, 37F) adult athletes, exercising on average 9.1±5.0 hours a week and 5.0±1.8 times a week, were included. REE was measured using indirect calorimetry (Vmax Encore n29), FFM and FM were measured using air displacement plethysmography. Multiple linear regression analysis was used to develop a new FFM-based and weight-based REE predictive equation. The percentage accurate predictions (within 10% of measured REE), percentage bias, root mean square error and limits of agreement were calculated.

**Results:**

The Cunningham equation and the new weight-based equation 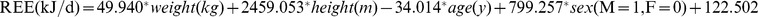 and the new FFM-based equation 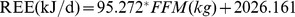 performed equally well. De Lorenzo's equation predicted REE less accurate, but better than the other generally used REE predictive equations. Harris-Benedict, WHO, Schofield, Mifflin and Owen all showed less than 50% accuracy.

**Conclusion:**

For a population of (Dutch) recreational athletes, the REE can accurately be predicted with the existing Cunningham equation. Since body composition measurement is not always possible, and other generally used equations fail, the new weight-based equation is advised for use in sports nutrition.

## Introduction

An optimal balance between energy intake and energy expenditure is crucial for athlete performance. Determining resting energy expenditure (REE) is essential for nutrition and exercise professionals in providing nutritional advice for athletes, since REE contributes to 60–70% of daily energy expenditure [1]. REE is the maintenance energy cost of the body in rest under steady state conditions. This is different from the minimal energy cost. Energy expenditure can for example be lower during sleep or during undernutrition [2]. REE can be measured through indirect calorimetry or estimated using REE predictive equations. The gold standard to determine the REE is measurement by indirect calorimetry. However, nutrition and exercise professionals do not usually have the necessary equipment. Moreover, indirect calorimetry [3] is expensive and requires trained personnel to guarantee the reliability. Therefore, predictive equations are most often used to estimate the REE.

In general, REE predictive equations can be considered population specific [4]–[8]. Because of their relatively high fat free mass (FFM), REE in athletes is underestimated by the majority of the existing equations [9]. Only a few studies have validated predictive equations in a group of athletes [10], [11]. Thompson and Manore [11] concluded that the Cunningham equation was the most accurate equation, while LaForgia et al. [10] indicated the equation of De Lorenzo as the most accurate predictive equation. However, it should be noted that both studies investigated a different set of equations.

Cunninghams equation was established using 239 healthy adult subjects from the studies of Harris and Benedict [12], but they excluded 16 male subjects because they were identified as trained athletes. Moreover, the only factor included in the equation is lean body weight, but body composition measurements are not always possible in sports practice. Factors in De Lorenzo's equation are weight and height, which are better applicable in sports practice. Moreover, the De Lorenzo equation is actually based on athletes [13]. However, the De Lorenzo equation was developed using the REE from 51 male athletes exercising at least 3 hours/day. This equation may therefore only apply to (sub) elite athletes while it may less accurately predict the REE for recreational athletes, which represent a significant part of the society. Therefore, the aim of this study was to validate existing resting energy expenditure predictive equations (Table 1) and to develop 2 new equations, specifically for recreational athletes. One equation was developed using FFM, since the body composition of (recreational) athletes differs from that of the average population. In addition, an equation was developed without FFM, since this parameter cannot always be used in sport practice. It was hypothesized that a new equation specifically developed for recreational athletes would result in better estimations of the resting energy expenditure.

**Table 1:** Equations evaluated in this study.

Equation	Sample size	Remarks on population
**Cunningham** [17]	M = 120 F = 103	Healthy adults, trained athletes were excluded
**De Lorenzo** [13]	M = 51	Athletes, exercising at least 3 hours a day
**FAO** [21]	N = 11000	Mainly based on Schofield data, also including group mean values also
**FAO (height)** [21]	N = 11000	Mainly based on Schofield data, also including group mean values
**Harris-Benedict 1919** [21]	M = 136 F = 103	Mainly normal weight subject
**Harris-Benedict 1984** [28]	M = 168 F = 169	Mainly normal weight subject
**Mifflin** [18]	M = 251 F = 247	Including obese subjects
**Mifflin (FFM)** [18]	M = 251 F = 247	Including obese subjects
**Owen** [19], [20]	M = 60 F = 44	Including obese subjects
**Owen (FFM)** [19], [20]	M = 60 F = 44	Including obese subjects
**Schofield** [29]	M = 3575 F = 1239	47% Italian subjects
**Schofield (height)** [29]	M = 3575 F = 1239	47% Italian subjects

## Methods

**Figure 1:**
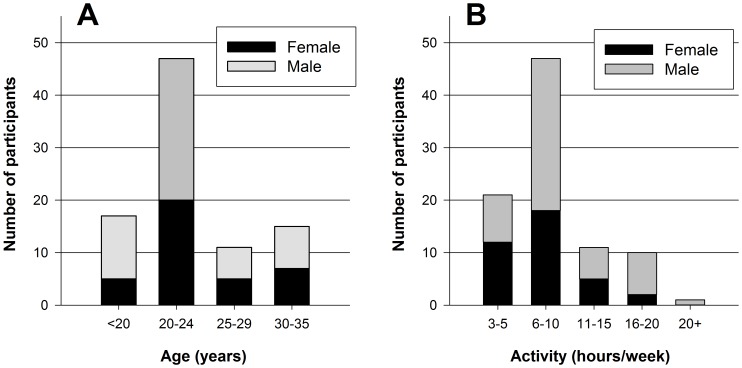
Description of the study participants (N = 90) for (A) age group and (B) activity level (hours per week).

103 athletes (age 18–35 y), who participated in sports for at least 3 hours a week and 2 times a week, participated in the study. Subjects were of self-reported stable weight, did not suffer from any metabolic disorders and did not take any medications affecting metabolic rate. They did not perform vigorous exercise within 12 hours before the measurement, neither did they consume food or beverages (except water) within 4 hours before the measurement. Subjects were at least 30 minutes at rest in the laboratory before the start of the measurement. 9 participants were excluded from analysis because they were in the ovulation phase of the menstrual cycle [14] and 4 were excluded because their Respiratory Exchange Ratio (RER) was below 0.7 or above 1.0 [15]. Finally, 90 athletes (Characteristics: Table 2; Sport specifications: Table 3; Age and Activity level: Figure 1) were included for validation and development of REE predictive equations. Mean (±SD) age was 23.2±4.8 year and mean REE was 7.68±1.16 MJ/day (1837±278 kcal/day). The training hours were 9.1±5.0 hours/week and the training frequency spent on sports was 5.0±1.8 times/week. Written informed consent was obtained from all subjects and the study was approved by the medical ethical committee of the VU University Medical Center (2009/302).

**Table 2:** Subjects characteristics: mean ± standard deviation [range].

	Male (N = 53)		Female (N = 37)		Total (N = 90)	
**Age (y)**	23.1±4.7	[18–35]	23.5±5.0	[18–35]	23.2±4.8	[18–35]
**Height (m)**	1.82±.09	[1.63–2.05]	1.72±.05	[1.61–1.84]	1.78±.09	[1.61–2.05]
**Weight (kg)**	75.7±7.8	[62.1–100.3]	62.6±6.6	[52.8–78.3]	70.3±9.7	[52.8–100.3]
**BMI (kg/m^2^)**	22.8±2.0	[18.6–27.0]	21.3±1.9	[17.9–26.7]	22.2±2.1	[17.9–27.0]
**FFM (%)**	88.3±4.9	[73.7–98.5]	78.4±4.7	[67.8–88.8]	84.3±6.8	[67.8–98.5]
**FM (%)**	11.7±4.9	[1.5–26.3]	21.6±4.7	[11.2–32.2]	15.7±6.8	[1.5–32.2]
**REE (kcal/d)**	2007±206	[1562–2551]	1594±162	[1111–1832]	1837±278	[1111–2551]
**REE (MJ/d)**	8.40±0.86	[6.53–10.67]	6.67±0.68	[4.65–7.67]	7.69±1.16	[4.65–10.67]
**Activity (h/week)**	10.0±5.4	[3.0–28.0]	7.9±4.0	[3.0–18.0]	9.1±5.0	[3.0–28.0]
**Activity (#/week)**	5.3±1.8	[2]–[12]	4.5±1.7	[2]–[9]	5.0±1.8	[2]–[12]

BMI: Body mass index, FFM: Fat free mass, FM: Fat mass, REE: Resting energy expenditure.

**Table 3:** Description of sports activity.

	Male (N = 53)	Female(N = 37)	Total (N = 90)
**Athletics Long distance**	5	9	14
**Athletics Sprint**	3	2	5
**Cycling**	6	4	10
**Gymnastics**	4	5	9
**Fitness**	3	5	8
**Rowing/canoeing**	7	5	12
**Swimming**	2	1	3
**Team sports** *	17	4	21
**Remaining sports** **	6	2	8

* Hockey, korfball, soccer, volleyball.

** Dancing, martial arts, skating, tennis.

REE was calculated by the manufacturer’s software, using oxygen consumption and carbon dioxide production measured by indirect calorimetry with a ventilated hood system (Vmax Encore n29; Viasys Healthcare, Houten, Netherlands). Calibration of the equipment with 2 different standard gases and 1 standard volume was performed on a daily basis, before starting the measurements. Additionally, an automatic recalibration of the system was done every 5 minutes. The subjects remained lying down but awake. The measurement took 30 minutes. Only steady state periods of measurement were selected according to the procedures for the ventilated hood system (<10% CV). The first 5 minutes of the measurements were discarded.

Body weight, FFM, and fat mass (FM) were determined using air displacement plethysmography (BODPOD, Life Measurement Inc, Concord, CA). Total lung volume was measured (31 were estimated) with the BODPOD system to correct total body volume. The BODPOD was calibrated on a daily basis directly before use. Height was measured by using a stadiometer (Seca 222; Seca; Hamburg; Germany).

Statistical analysis was performed using SPSS 20. Multiple linear regression analysis was used to develop 2 new REE predictive equations. Firstly, a stepwise regression approach was used with the variables FFM, FM, age, sex and a constant to verify a possible FFM-based REE equation. Weight-based equations are usually most successful when predictors of active cell mass are entered, being body weight, height, age and sex [16]. Therefore, these variables and a constant were used in a regression analysis. In both regression analyses, variables were included when the p-value for the F-test was smaller than 0.05.

The predicted REE from the 2 developed equations and from other commonly used equations (Table 4) were compared with the measured REE. Firstly, accuracy was calculated as the percentage of accurate REE predictions (within 10% of the measured REE). Secondly, the bias was calculated as the relative deviation (%) of the predicted REE from the measured REE. Thirdly, the root mean squared error (RMSE) was calculated as the expected absolute deviation (MJ/d) of the predicted REE from the measured REE. Lastly, limits of agreement were calculated as the 95% confidence interval of the bias.

**Table 4:** Resting energy expenditure prediction equations given in their original unit (kcal/day, except Schofield (MJ/day)).

Name	Equation
**Cunningham**	22(FFM) +500
**De Lorenzo**	9(wt) +1170(ht) –857
**FAO**	
Male (18–30 y)	15.3(wt) +679
Male (30–60 y)	11.6(wt) +879
Male (>60 y)	13.5(wt) +487
Female (18–30 y)	14.7(wt) +496
Female (30–60 y)	8.7(wt) +829
Female (>60 y)	10.5(wt) +596
**FAO** (height)	
Male (18–30 y)	15.4(wt) –27(ht) +717
Male (30–60 y)	11.3(wt) –16(ht) +901
Male (>60 y)	8.8(wt) + 1128(ht) –1071
Female (18–30 y)	13.3(wt) +334(ht) +35
Female (30–60 y)	8.7(wt) –25(ht) +865
Female (>60 y)	9.2(wt) + 637(ht) –302
**Harris-Benedict 1919**	
Male	13.75(wt) +500.33(ht) –6.76(age) +66.47
Female	9.56(wt) +184.96(ht) –4.68(age) +655.10
**Harris-Benedict 1984**	
Male	13.40(wt) +479.9(ht) –5.68(age) +88.36
Female	9.25(wt) +309.8(ht) –4.33(age) +477.59
**Mifflin**	9.99(wt) +625(ht) –4.92(age) +166(gender) –161
**Mifflin **(FFM)	19.7(FFM) +413
**Owen**	
Male	10.2(wt) +879
Female	7.18(wt) +795
**Owen** (FFM)	
Male	22.3(FFM) +290
Female	19.7(FFM) +334
**Schofield**	
Male (18–30 y)	63(wt) +2896*
Male (30–60 y)	48(wt) +3653*
Male (>60 y)	49(wt) +2459*
Female (18–30 y)	62(wt) +2036*
Female (30–60 y)	34(wt) +3538*
Female (>60 y)	38(wt) +2755*
**Schofield **(height)	
Male (18–30 y)	63(wt) –42(ht) +2953*
Male (30–60 y)	48(wt) –11(ht) +3670*
Male (>60 y)	49(wt) +4068(ht) –3491*
Female (18–30 y)	62(wt) +1148(ht) +411*
Female (30–60 y)	34(wt) +6(ht) +3530*
Female (>60 y)	38(wt) +1917(ht) +74*

FFM: Fat free mass (kg); wt: weight (kg); ht: height (m); age (y); gender (0: female, 1: male).

* Equation for values in MJ/day.

## Results

Using the previously described regression analyses, the following new equations were obtained (in kJ and kcal) for weight-based:



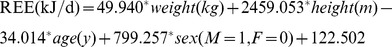
 or,
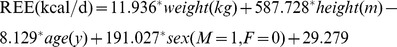
and FFM-based:



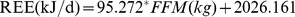
or,



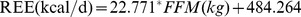



**Figure 2:**
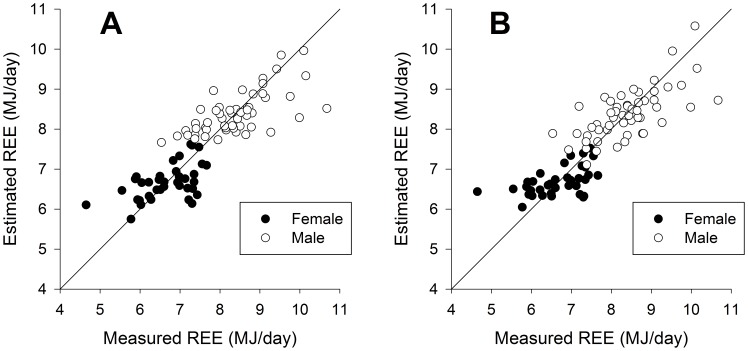
Predicted REE (MJ/day) plotted against REE (MJ/day) measured by indirect calorimetry for male (open dot) and female (filled dot). Graphs represent (A) the new weight-based and (B) the new FFM-based equation. The solid line is the line of identity.

The weight-based equation for resting energy expenditure included body weight (kilograms), body height (meters), age (years), sex (value 1 for male, value 0 for female) and a constant. The fat free mass based equation only included fat free mass (kilograms) and a constant. The predicted REE values are plotted against measured REE values (Figure 2).

**Figure 3:**
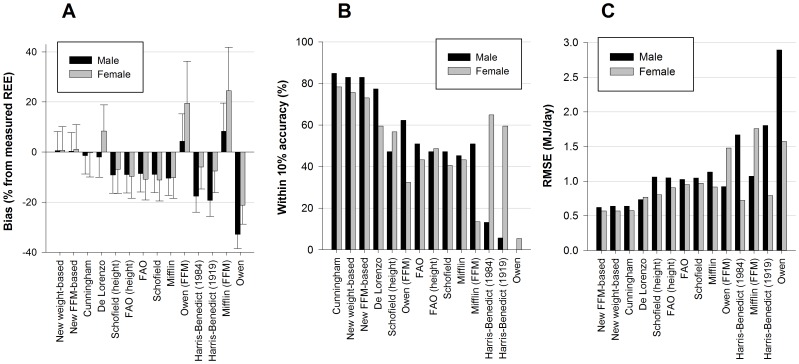
Outcome measures for the REE predictive equations for male (N = 53) and female (N = 37). The graphs represent (**A**) Percentage bias (±SD), (**B**) percentage accurate REE predictions and (**C**) Root mean squared error (MJ/day).

The accuracy for all equations is depicted in Figure 3B. The Cunningham equation resulted in the highest percentage accurate predicted equations (M 84.9%; F 78.4%), followed by the weight-based equation (M 83.0%; F 75.7%) and the FFM-based equation (M 83.0%; F 72.3%). The De Lorenzo equation for elite male athletes (M 77.4%; F 59.5%) had an inferior accuracy compared to the weight-based equation. All other, generally used equations showed less than 50% accuracy.

Equal to the accuracy, lowest bias was observed for Cunningham, both new equations and De Lorenzo (Figure 3A). The low bias for De Lorenzo's equation was based on opposite bias (male: negative, female: positive). Since this is not a cross-validation study, the bias for the new equations approached zero.

The FFM-based equation resulted in the lowest RMSE (M 0.62 MJ/d; F 0.57 MJ/d), and Cunningham's and the weight-based equation resulted in a comparable RMSE (Figure 3C). The RMSE for De Lorenzo was higher (M 0.74 MJ/d; F 0.77 MJ/d). The generally used equations all resulted in RMSE larger than 0.95 MJ/day.

**Figure 4:**
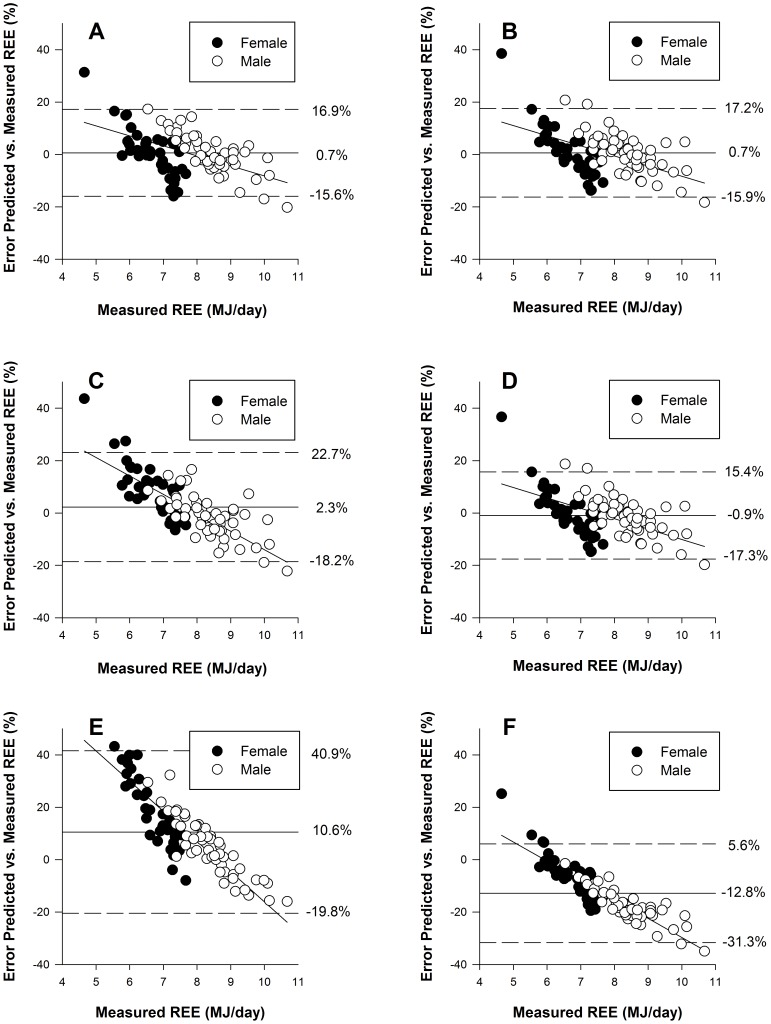
Limits of agreement analysis (bias: vertical solid line; 95% confidence interval: vertical dashed lines) for REE predictive equations versus measured REE (MJ/day) for male (open dot) and female (filled dot). Graphs represent equations of (**A**) new weight-based; (**B**) new FFM-based; (**C**) De Lorenzo; (**D**) Cunningham; (**E**) Owen FFM and (**F**) Harris-Benedict 1984.

In Figure 4 the errors are plotted against the individual measured REE for 6 equations. A negative proportional bias could be observed for all equations, meaning the equations tended to underestimate a higher REE.

## Discussion

The purpose of this study was to validate existing predictive equations for REE and to develop and validate 2 new equations, specifically for recreational athletes. This study shows that the new FFM-based equation provides the best prediction of REE in recreational athletes. The weight-based equation and Cunningham's equation [17] performed equally well. De Lorenzo's equation [13] performed less, and can be improved by the new weight-based equation. The other generally used REE predictive equations have low accuracy in recreational athletes.

Although the Cunningham equation was developed in a general population, this equation performed better than the already existing equations. This suggests that FFM is an important predictor of REE for a population of recreational athletes. However, the FFM-based equations from Mifflin [18] and Owen [19], [20] did perform worse than the new weight-based equation and De Lorenzo's equation [13], and not better than the other non-FFM equations. The difference of those equations with Cunningham [17] and the new FFM-based equation might be explained by the population that was used to develop the equations. Mifflin [18] and Owen [19], [20] used lean and obese subjects. The mean BMI in Mifflin's study was 27.5±4.1 kg/m^2^ for male and 26.2±4.9 kg/m^2^ for female, with a maximum of 42 kg/m^2^. In Owen's study, the BMI for male was 28.2±7.5 kg/m^2^ and 27.8±8.6 kg/m^2^ for female, with a maximum of 58.7 kg/m^2^. Therefore, the better performance of the new FFM-based and Cunningham's equation over the weight and/or height based equations might result from the different body composition of (recreational) athletes compared with a general population.

De Lorenzo's equation [13] is the only equation which is actually based on a population of athletes. The accuracy was higher than the other generally used equations, but lower than Cunningham and the new developed equations. The participants in the study of De Lorenzo exercised at least 3 hours/day. The equation includes body weight and height and probably these factors do not predict resting energy expenditure well in recreational athletes due to a substantial different body composition. This emphasises the role of the specificity of the population. Other studies have pointed out that the accuracy of a prediction equation not only depends on activity level. For example, Henry [2] described the inaccuracy of the FAO equations [21] for many communities. The population used to develop the FAO equations consisted of a disproportional number of Italians, who seem to have a higher basal metabolic rate then people living in the tropical area, China or Australia. Another example of the specific accuracy of REE prediction equation is depicted by Weijs [5], who showed differences between US and Dutch obese adults. As a consequence of the specificity of a REE equation, a researcher always has to deal with a trade-off between internal and external validity (i.e. the more homogeneous the population, the more accurate the REE prediction but the less applicable to a heterogeneous population). Therefore, validation of the new equations in other cohorts is needed.

This study has some major limitations. Firstly, participants were instructed to not perform vigorous exercise within 12 hours before the measurement. However, Compher et al. [15] advised to perform no physical activity 14 hours prior to indirect calorimetry measurement to have energy expenditure returned to baseline levels. Physical activity will increase REE after exercise [22], [23]. The time needed for REE to return to normal is called recovery time, which is dependent on the type of activity, intensity, duration and physical exercise level. In trained individuals, like in this study, recovery time is shorter than in untrained [15]. The study upon which the advice of 14 hours is based, included only 10 subjects [24]. Another small, but well designed study [25] used a 12 hour recovery time for different levels of activity, and showed no effect of physical activity on REE. Therefore, a period of 12 hours was selected for the present study.

Secondly, subjects were instructed to not eat within 4 hours prior to the measurement, while Compher et al. [15] recommended a minimal fast of 5 hours. However, they stated that for small meals a minimum fast of 4 hours is acceptable. Moreover, Weststrate [26], [27] showed that the thermic effect of food could be nearly completed within 4 hour. The mean period of fasting by our subjects was on average 9.1±4.6 hours. Therefore, it is assumed that the thermic effect of food was negligible in this study.

## Conclusions

In general, it is advised to use the REE prediction equation that is most specific for the target population. For a population of (Dutch) recreational athletes, the REE can accurately be predicted with the existing Cunningham equation. Since body composition measurement is not always possible, and other generally used equations fail, the new weight-based equation is advised for use in sports nutrition.

## Supporting Information

Dataset S1 **Contains raw data used for development and testing of the prediction equations.** The dataset contains information on gender; age; training volume; training frequency; weight; fat mass (%); fat mass (kg); fat free mass (kg); height; BMI; fat free mass (%) and measured REE (kJ) of the subjects.
(SAV)
